# Investigations of Heavy Metal Ion Sorption Using Nanocomposites of Iron-Modified Biochar

**DOI:** 10.1186/s11671-017-2201-y

**Published:** 2017-06-30

**Authors:** D. Kołodyńska, J. Bąk, M. Kozioł, L. V. Pylychuk

**Affiliations:** 1 Department of Inorganic Chemistry, Faculty of Chemistry, Maria Curie-Skłodowska University, M. Curie Skłodowska Sq. 2, 20-031 Lublin, Poland; 2 Department of Organic Technologies, New Chemical Syntheses Institute, Al.Tysiąclecia Państwa Polskiego 13A, 24-110 Puławy, Poland; 3 Nanomaterials Department, Chuiko Institute of Surface Chemistry of the National Academy of the Sciences of Ukraine, General Naumov Str., Kyiv, 03-164 Ukraine

**Keywords:** Nanocomposites, Magnetic biochar, Heavy metal ions, Iron modification, Sorption

## Abstract

Magnetic biochar nanocomposites were obtained by modification of biochar by zero-valent iron. The article provides information on the impact of contact time, initial Cd(II), Co(II), Zn(II), and Pb(II) ion concentrations, dose of the sorbents, solution pH and temperature on the adsorption capacity. On the basis of experiments, it was found that the optimum parameters for the sorption process are phase contact time 360 min (after this time, the equilibrium of all concentrations is reached), the dose of sorbent equal to 5 g/dm^3^, pH 5 and the temperature 295 K. The values of parameters calculated from the kinetic models and isotherms present the best match to the pseudo second order and Langmuir isotherm models. The calculated thermodynamic parameters *∆H* ^0^, *∆S* ^0^ and *∆G* ^0^ indicate that the sorption of heavy metal ions is an exothermic and spontaneous process as well as favoured at lower temperatures, suggesting the physical character of sorption. The solution of nitric acid(V) at the concentration 0.1 mol/dm^3^ was the best acidic desorbing agent used for regeneration of metal-loaded magnetic sorbents. The physicochemical properties of synthesized composites were characterized by FTIR, SEM, XRD, XPS and TG analyses. The point characteristics of the double layer for biochar pH_PZC_ and pH_IEP_ were designated.

## Background

The growing amount of agricultural wastes which is landfilled or burned causes groundwater contamination or air pollution [1]. These wastes which include hazelnut husks [2]; wood, bark and corn straw [3, 4]; rice husks and empty fruit brunch [5]; potato peel [6] and sugar beet tailing [7] are the raw materials for the production of biochar. In the pyrolysis process, properly selected conditions allow to obtain low-cost sorbents of high porosity and suitable surface area [8, 9]. The addition of biochar to the soil increases its fertility because of its abundant organic matter [10]. Biochar is also used as a sorbent for the removal of heavy metal ions: Cu(II), Cd(II) [11, 12], Cr(VI), Pb(II) [13], Ni(II) [14] and others.

Application of nanocomposites of iron-modified biochar can overcome the difficulties associated with separation of biochar after sorption. These nanocomposites have magnetic properties so that when the external field is applied, they can be removed from the solutions [15]. Fe, Fe_2_O_3_ and Fe_3_O_4_ are magnetic particles used in two types of modification of biochar by pyrolysis at high temperatures or chemical coprecipitation [16–23]. Zhang et al. [16] obtained magnetic biochar by pretreatment of biomass (cotton wood) in a ferric chloride solution and then subjecting it to a pyrolysis at a temperature 873 K for 1 h. Biochar/γ-Fe_2_O_3_ demonstrated the ability of As(V) ion sorption from aqueous solutions. Three novel magnetic biochars were synthesized by Chen et al. [17] by chemical co-precipitation in a solution of ferrous chloride and ferric chloride (molar ratio 1:1) on biomass (orange peels) and then pyrolysis at different temperatures 523, 673, and 973 K. Magnetite biochar (obtained at 523 K) indicates the increase of the sorption percentage of phosphates from 7.5% (for non-magnetic biochar) to 67.3%. In addition, the resulting sorbent is capable of simultaneous removal of phosphates and organic impurities which is important because these compounds coexist in wastewaters. Wang et al. [18] investigated the regeneration of Pb-loaded magnetic biochar. This sorbent was prepared by mixing biochar (obtained from eucalyptus leaf residue) with FeCl_3_ and FeSO_4_ solutions and the addition of NaOH up to the pH value 10–11. The use of EDTA-2Na as a desorbing agent gives the yield of 84.1% which confirms that the magnetic biochar can be a sorbent of multi-use. Zero-valent iron-impregnated biochar was obtained by Devi and Saroha [21] and was used for the removal of pentachlorophenol from effluents. It was found that the best sorption parameters were obtained by magnetic biochar at the molar ratio FeSO_4_:NaBH_4_ = 1:10 and the sorption percentage was 80.3%.

Zero-valent iron-coated biochar is characterized by high reactivity and high affinity for the impurities in aqueous solutions of the organic compounds: pentachlorophenol [22] and trichloroethylene [23] as well as the heavy metal ions As(V) [24], Cr(VI) [10] and Pb(II) [25].

In this paper, two types of magnetic biochar were used to test the ability of heavy metal ion capture. For modifications, FeSO_4_ as a source of iron and NaBH_4_ as a reducing agent at the different molar ratios of FeSO_4_ to NaBH_4_ 1:1 and 1:2 were used. The obtained sorbents were designated as MBC1 and MBC2, respectively. To understand the mechanism of heavy metal ions Cd(II), Co(II), Zn(II) and Pb(II) adsorption on MBC1 and MBC2, effects of the sorbent dose, phase contact time, initial concentration, solution pH and temperature were investigated. To describe the kinetics and equilibrium adsorption, the pseudo first order, pseudo second order and intraparticle diffusion kinetic models as well as the adsorption isotherms of Langmuir and Freundlich models were applied. Fourier transform infrared spectroscopy, scanning electron microscopy, X-ray photoelectron spectra and TG/DTG curves were used to characterize the physicochemical properties of two modifications. The point of zero charge pH_PZC_ and the isoelectric point pH_IEP_ are also determined. Additionally, the efficiency of sorbent regeneration using HNO_3_ at different concentrations was determined.

## Methods

### Preparation of Sorbents

A dry sorbent biochar used in the experiment comes from Coaltec Energy, USA Inc., and is produced in the gasification process. Gasification involves heating the biomass in an oxygen-free atmosphere. The result is a biochar carbon-rich sorbent [26].

Zero-valent iron-coated biochars (magnetic ones) were prepared by dissolving FeSO_4_·7H_2_O (0.18 mol/dm^3^) in 100 cm^3^ of distilled water while stirring the solution and adding 5 g of biochar. The NaBH_4_ solution results in a reduction of Fe(II) to Fe(0), and it is added dropwise into the suspension while stirring at 1000 rpm for 30 min under room temperature. Then the nanocomposite was filtered and washed as well as dried in the oven. For the molar ratio of FeSO_4_ to NaBH_4_ = 1:1, 4.96 g of FeSO_4_ and 0.68 g of NaBH_4_ were used and the sorbent was denoted as MBC1. For the second modification, for MBC2, the same amounts of FeSO_4_ and 1.36 g of NaBH_4_ were applied.

### Chemicals

The chemicals used in the experiment were of analytical grade and purchased from Avantor Performance Materials (Poland). The stock solutions of Cd(II), Co(II), Zn(II) and Pb(II) ions at a concentration 1000 mg/dm^3^ were prepared by dissolving the appropriate amounts of salts Cd(NO_3_)_2_·4H_2_O, CoCl_2_·6H_2_O, ZnCl_2_ and Pb(NO_3_)_2_ in distilled water; 1 mol/dm^3^ of HCl and/or 1 mol/dm^3^ of NaOH were used for pH adjustment.

### Sorption and Kinetic Studies

These experiments were carried out in 100 cm^3^ conical flasks with 0.1 g of sorbents and 20 cm^3^ of solutions at the concentrations 50–200 mg/dm^3^, at the phase contact times from 0 to 360 min, at pH 5 and at 295 K. Then after shaking, the solutions were filtered and analysed for residual heavy metal ion concentrations by means of the atomic absorption spectroscopic methods. Finally, the equilibrium sorption capacity *q*_*e*_ [mg/g] was calculated according to the equation

1 $$ {\mathit{\mathsf{q}}}_{\mathit{\mathsf{e}}}=\frac{\left({\mathit{\mathsf{C}}}_{\mathsf{0}}-{\mathit{\mathsf{C}}}_{\mathit{\mathsf{e}}}\right)\mathit{\mathsf{V}}}{\mathit{\mathsf{m}}} $$

where *C*_0_ and *C*_*e*_ [mg/dm^3^] are the initial and equilibrium concentrations, *V* [dm^3^] is the volume of the metal ion solution, and *m* [g] is the mass of magnetic biochars.

To estimate the effect of dose on the Cd(II) ion sorption on two types of sorbents, 0.1 g of MBC1 and MBC2 and the 20 cm^3^ (5 g/dm^3^) of 100 mg/dm^3^ Cd(II) ion solution were used. The investigations were carried out for the doses of sorbents 5, 7.5 and 10 g/dm^3^, at pH 5, shaken mechanically at 180 rpm on a laboratory shaker at 295 K for 360 min. After shaking, the solutions were filtered and the contents of Cd(II) ions were measured.

The tests of the pH effect on the above-mentioned heavy metal ion sorption were carried out for MBC1 and MBC2. The amounts of the sorbents and the volumes of the solutions are the same as these mentioned above. The samples were shaken at a concentration of 100 mg/dm^3^ for 360 min and in the pH range 2–6.

The studies of the equilibrium sorption isotherm were conducted applying the same procedure as in kinetic investigations. MBC1 and MBC2 were in contact with the ion solutions at the concentrations 50–600 mg/dm^3^ for 360 min, at 180 rpm, at pH 5 and at 295 K. The sorption of Cd(II) on MBC1 and MBC2 was also studied as a function of temperature. Tests were carried out at 295, 315 and 335 K for the same solution concentrations as those in the adsorption tests. The thermodynamic parameters were calculated using the following equations:

2 $$ \mathit{\mathsf{\varDelta}}{\mathrm{G}}^{\mathrm{o}}=-\mathit{\mathsf{R}}\mathit{\mathsf{T}} \ln {\mathit{\mathsf{K}}}_{\mathit{\mathsf{d}}} $$

3 $$ \mathit{\mathsf{\varDelta}}{\mathit{\mathsf{G}}}^{\mathit{\mathsf{o}}}=\mathit{\mathsf{\varDelta}}{\mathit{\mathsf{H}}}^{\mathit{\mathsf{o}}}-\mathit{\mathsf{T}}\mathit{\mathsf{\varDelta}}{\mathit{\mathsf{S}}}^{\mathit{\mathsf{o}}} $$

4 $$ {\mathit{\mathsf{K}}}_{\mathit{\mathsf{d}}}=\frac{{\mathit{\mathsf{C}}}_{\mathit{\mathsf{s}}}}{{\mathit{\mathsf{C}}}_{\mathit{\mathsf{e}}}} $$

5 $$ \ln {\mathit{\mathsf{K}}}_{\mathit{\mathsf{d}}}=\frac{\mathit{\mathsf{\varDelta}}{\mathit{\mathsf{H}}}^{\mathit{\mathsf{o}}}}{\mathit{\mathsf{R}}\mathit{\mathsf{T}}}+\frac{\mathit{\mathsf{\varDelta}}{\mathit{\mathsf{S}}}^{\mathit{\mathsf{o}}}}{\mathit{\mathsf{R}}} $$

where *C*_*s*_ [mg/g] and *C*_*e*_ [mg/g] are the sorption capacities in the adsorbent and adsorbate phases, *∆G* ^0^ [kJ/mol] is the standard free energy changes, *R* is the gas constant [J/mol K], *T* is the temperature [K], *K*_*d*_ is the distribution coefficient, *∆H* ^0^ is the change of enthalpy [kJ/mol], and *∆S* ^0^ is the change of entropy [kJ/mol].

Efficiency of the sorbent regeneration was tested using distilled water and HNO_3_ at the concentrations 0.1, 0.5, 1.0, 1.5, 2.0 and 5.0 mol/dm^3^. After Cd(II) ion sorption at 100 mg/dm^3^ (pH 5, shaking speed 180 rpm, temperature 295 K), the Cd-loaded MBC2 samples were dried, weighed and shaken with 20 cm^3^ water or HNO_3_ at different concentrations for 360 min. The desorption yield was calculated as

6 $$ \%\mathit{\mathsf{Desorption}}=\frac{{\mathit{\mathsf{C}}}_{\mathit{\mathsf{d}}\mathit{\mathsf{e}}\mathit{\mathsf{s}}}}{{\mathit{\mathsf{C}}}_{\mathsf{0}}-{\mathit{\mathsf{C}}}_{\mathit{\mathsf{e}}}}\mathsf{100}\% $$

where *C*_des_ [mg/dm^3^] is the amount of metal ions in solution after regeneration.

### Apparatus and Analysis

Experiments were carried out by shaking the samples by means of the laboratory shaker type 358A (Elpin Plus, Poland). The pH values of samples after the sorption were measured using a pHmeter pHM82 (Radiometer, Copenhagen). Subsequently, the amounts of heavy metal ions were determined using an atomic absorption spectrometer AAS (Spectr AA 240 FS, Varian) at 228.8 nm for Cd(II), 240.7 nm for Co(II), 213.9 nm for Zn(II) and 217.0 nm for Pb(II).

The FTIR spectra of MBC1 and MBC2 were registered by means of a Cary 630 FTIR spectrometer (Agilent Technologies) before and after Co(II) sorption. They were obtained in the range 650–4000 cm^−1^.

The surface morphology of nanocomposites of iron-modified biochar was observed using the scanning electron microscope SEM (Quanta 3D FEG, FEI).

X-ray diffraction (XRD) was obtained using the X-ray diffractometry PANalytical (Empyrean, Netherlands).

X-ray photoelectron spectra (XPS) of MBC2 after the Cd(II) sorption were obtained using the UHV multi-chamber analytical system (Prevac, Poland).

The thermogravimetric (TG) and derivative thermogravimetric (DTG) analyses for MBC1 and MBC2 were made by means of TA Instruments Q50 TGA in nitrogen atmosphere before and after heavy metal ion sorption.

The zeta potential of biochar was determined by electrophoresis using Zetasizer Nano-ZS90 by Malvern. The measurements were performed at 100 ppm concentration ultrasonication of the suspension. As a background electrolyte, NaCl solution was used at the concentrations 0.1, 0.01 and 0.001 mol/dm^3^. The electrophoretic mobility was converted to the zeta potential in millivolts using the Smoluchowski equation.

Surface charge measurements were performed simultaneously in the suspension of the same solid content to maintain the identical conditions of the experiments in a thermostated Teflon vessel at 298 K. To eliminate the influence of CO_2_, all potentiometric measurements were performed in nitrogen atmosphere. The pH values were measured using a set of glass REF 451 and calomel pHG201-8 electrodes with the Radiometer assembly. The surface charge density was calculated from the difference of the amounts of added acid or base to obtain the same pH value of suspension as for the background electrolyte. The density of biochar surface charge was determined using the “titr_v3” programme. Comparison of the titration curve of the metal oxide suspension of the same ionic strength is used to determine the surface charge density of metal oxide. The surface charge density is calculated from the ratio of the volume of acid and base added to the suspension in order to obtain the desired pH value:

7 $$ {\mathit{\mathsf{\sigma}}}_{\mathsf{0}}=\frac{\mathit{\mathsf{\varDelta VCF}}}{{\mathit{\mathsf{S}}}_{\mathit{\mathsf{w}}}\mathit{\mathsf{m}}} $$

where Δ*V* is the ratio of the volume of acid and base added to the suspension in order to obtain the desired pH value, *C* [mol/dm^3^] is the concentration of acid/base, *F* [9.648 × 10^4^ C mol^−1^] is the Faraday constant, *m* [g] is the mass of metal oxide, and *S*_*w*_ is the specific surface area of metal oxide.

## Results and Discussion

### Adsorption Kinetics

**Fig. 1:**
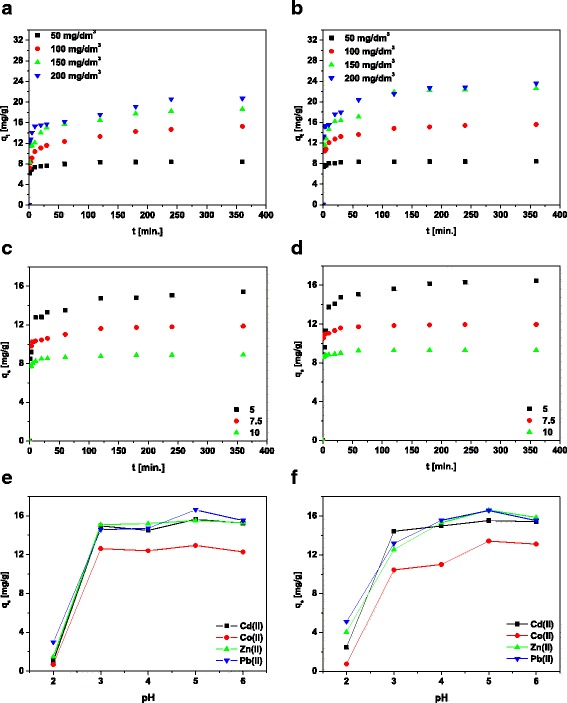
Effect of the phase contact time on Cd(II) adsorption on **a** MBC1 and **b** MBC2, effect of dose of **c** MBC1 and **d** MBC2 on Cd(II) sorption and effect of pH on heavy metal ion sorption on **e** MBC1 and **f** MBC2

In order to estimate the sorption capacity of MBC1 and MBC2, it is important to determine the equilibrium time for maximum removal of heavy metal ions. Therefore, studies were performed with various initial concentrations from 50 to 200 mg/dm^3^ and in the contact time range of 1–360 min. Following from Fig. 1a, b, the sorption capacities of metal ions rose sharply at short contact time and slowed gradually as the state of equilibrium was reached. Due to the large number of free active sites on the surface of the magnetic biochar in the initial stage, sorption occurs rapidly [27]. The equilibrium is achieved faster for lower initial concentrations, after approximately 60 min for the Cd(II) ion concentration 50 mg/dm^3^ and slower for higher initial concentration, for instance after approximately 240 min for the concentration 200 mg/dm^3^.

Capacity equilibria increased with the increasing contact time and initial concentration and are equal to 8.40, 15.29, 18.65, and 20.65 mg/g for the Cd(II) at concentrations 50, 100, 150, and 200 mg/dm^3^, respectively, for MBC1 and 8.41, 15.63, 22.63 and 23.55 mg/g, respectively, for MBC2. In addition, it can be concluded that the modification with a higher content of a reducing agent has a higher value of *q*_*e*_. For Co(II), Zn(II) and Pb(II) ions, the same relationships were found. The values of equilibrium capacities contained in Tables 1 and 2 permit to establish of a series of affinity of heavy metal ions for nanocomposites of iron-modified biochar Pb(II) > Zn(II) > Cd(II) > Co(II).

**Table 1:** Parameters for various adsorption kinetic models for Cd(II), Co(II), Zn(II) and Pb(II) sorption on MBC1

Parameters											
*C*_0_ [mg/dm^3^]	*q*_exp_	PFO			PSO				IPD		
		$\log \left({\mathit{\mathsf{q}}}_{{}_{\mathsf{1}}}-{\mathit{\mathsf{q}}}_{{}_{\mathit{\mathsf{t}}}}\right)= \log \left({\mathit{\mathsf{q}}}_{{}_{\mathsf{1}}}\right)-\frac{\mathit{\mathsf{k}}{}_{{}_{\mathsf{1}}}\mathit{\mathsf{t}}}{\mathsf{2.303}}$			$\frac{\mathit{\mathsf{t}}}{{\mathit{\mathsf{q}}}_{\mathit{\mathsf{t}}}}=\frac{\mathsf{1}}{{\mathit{\mathsf{k}}}_{\mathsf{2}}{\mathit{\mathsf{q}}}_{\mathsf{2}}^{\mathsf{2}}}+\frac{\mathit{\mathsf{t}}}{{\mathit{\mathsf{q}}}_{\mathsf{2}}}$				${\mathit{\mathsf{q}}}_{\mathit{\mathsf{t}}}={\mathit{\mathsf{k}}}_{\mathit{\mathsf{i}}}{\mathit{\mathsf{t}}}^{\mathsf{1}/\mathsf{2}}+\mathit{\mathsf{C}}$		
		*q*_1_	*k*_1_	*R* ^2^	*q*_2_	*k*_2_	*h*	*R* ^2^	*k*_*i*_	*C*	*R* ^*2*^
Cd(II)											
50	8.40	1.40	0.017	0.945	8.43	0.067	4.773	1.000	0.358	6.079	0.878
100	15.29	6.08	0.010	0.966	15.25	0.009	2.070	0.998	1.126	6.389	0.953
150	18.65	7.14	0.012	0.958	18.70	0.009	2.985	0.998	1.423	7.914	0.851
200	20.65	8.33	0.014	0.820	20.70	0.007	2.801	0.996	1.033	11.3146	0.889
Zn(II)											
50	8.82	0.81	0.024	0.913	8.84	0.165	12.892	1.000	0.339	6.946	0.820
100	15.87	6.32	0.015	0.977	16.02	0.010	2.595	0.999	0.732	8.571	0.886
150	20.41	7.19	0.010	0.981	20.44	0.007	2.814	0.997	0.610	12.409	0.799
200	27.59	10.09	0.006	0.866	26.76	0.005	3.326	0.993	1.523	12.854	0.964
Co(II)											
50	7.71	2.97	0.011	0.970	7.71	0.019	1.116	0.998	0.390	3.843	0.922
100	12.12	6.55	0.015	0.950	12.29	0.009	1.314	0.998	0.662	4.728	0.968
150	14.84	7.82	0.012	0.928	14.95	0.006	1.299	0.993	0.254	7.044	0.979
200	17.32	10.70	0.009	0.935	17.36	0.003	0.947	0.979	0.688	6.110	0.735
Pb(II)											
50	8.74	0.02	0.061	0.586	8.74	3.780	288.864	1.000	0.008	8.704	0.575
100	16.92	0.07	0.013	0.892	16.92	0.882	252.606	1.000	0.006	16.849	0.692
150	23.75	0.02	0.008	0.447	23.74	4.056	286.943	1.000	0.025	23.648	0.479
200	33.13	0.15	0.020	0.633	33.14	0.872	957.123	1.000	0.371	31.713	0.721

**Table 2:** Parameters for various adsorption kinetic models for Cd(II), Co(II), Zn(II) and Pb(II) sorption on MBC2

Parameters											
*C*_0_ [mg/dm^3^]	*q*_exp_	PFO			PSO				IPD		
		$\log \left({\mathit{\mathsf{q}}}_{{}_{\mathsf{1}}}-{\mathit{\mathsf{q}}}_{{}_{\mathit{\mathsf{t}}}}\right)= \log \left({\mathit{\mathsf{q}}}_{{}_{\mathsf{1}}}\right)-\frac{\mathit{\mathsf{k}}{}_{{}_{\mathsf{1}}}\mathit{\mathsf{t}}}{\mathsf{2.303}}$			$\frac{\mathit{\mathsf{t}}}{{\mathit{\mathsf{q}}}_{\mathit{\mathsf{t}}}}=\frac{\mathsf{1}}{{\mathit{\mathsf{k}}}_{\mathsf{2}}{\mathit{\mathsf{q}}}_{\mathsf{2}}^{\mathsf{2}}}+\frac{\mathit{\mathsf{t}}}{{\mathit{\mathsf{q}}}_{\mathsf{2}}}$				${\mathit{\mathsf{q}}}_{\mathit{\mathsf{t}}}={\mathit{\mathsf{k}}}_{\mathit{\mathsf{i}}}{\mathit{\mathsf{t}}}^{\mathsf{1}/\mathsf{2}}+\mathit{\mathsf{C}}$		
		*q*_1_	*k*_1_	*R* ^2^	*q*_2_	*k*_2_	*h*	*R* ^2^	*k*_*i*_	*C*	*R* ^*2*^
Cd(II)											
50	8.41	0.54	0.019	0.914	8.42	0.227	16.087	1.000	0.224	7.188	0.897
100	15.63	4.42	0.013	0.978	15.67	0.016	3.846	0.999	0.756	9.385	0.933
150	22.63	10.41	0.018	0.968	23.02	0.006	3.189	0.998	1.472	9.698	0.960
200	23.55	8.20	0.011	0.964	23.59	0.006	4.072	0.999	1.076	12.664	0.893
Zn(II)											
50	8.82	0.24	0.025	0.913	8.83	0.658	51.259	1.000	0.187	8.007	0.789
100	16.85	3.53	0.012	0.965	16.87	0.020	5.820	1.000	0.449	12.168	0.914
150	20.57	6.40	0.008	0.963	20.40	0.008	3.124	0.997	0.613	12.870	0.888
200	27.93	8.20	0.012	0.971	27.99	0.007	5.546	0.999	0.891	17.933	0.967
Co(II)											
50	8.12	1.68	0.016	0.938	8.15	0.055	3.675	1.000	0.452	5.113	0.981
100	12.84	6.40	0.010	0.940	12.82	0.007	1.187	0.994	0.621	5.207	0.847
150	15.24	7.99	0.011	0.991	15.36	0.005	1.292	0.995	0.387	6.984	0.974
200	18.30	8.72	0.007	0.917	17.96	0.005	1.525	0.992	0.370	8.150	0.965
Pb(II)											
50	8.74	0.02	0.004	0.656	8.73	2.335	178.025	1.000	0.003	8.704	0.982
100	16.93	0.01	0.007	0.705	16.93	5.530	158.227	1.000	0.003	16.905	0.666
150	23.75	0.04	0.008	0.920	23.74	1214	684.238	1.000	0.003	23.698	0.982
200	33.19	0.08	0.020	0.711	33.19	1.822	200.745	1.000	0.159	32.586	0.635

To describe the kinetics of heavy metal ion adsorption on magnetic sorbents, the pseudo first order (PFO), the pseudo second order (PSO), and the intraparticle diffusion (IPD) models were applied [28–30]. The kinetic parameters and correlation coefficients (*R* ^*2*^) are presented in Tables 1 and 2. According to the results of PFO model, the calculated values of equilibrium capacities were different compared to the experimental ones. The values of *R* ^*2*^ (>0.97) of PSO model indicate that this model seems to be the best to describe sorption process. In addition, the experimental values of *q*_*e*_ are similar to the theoretical ones. Moreover, the values of rate constants (*k*_*2*_) of PSO decrease with the increasing initial concentration of solutions from 0.067 to 0.007 g/(mg min) for MBC1.

### Effect of Dose

The relationship between two types of magnetic sorbents loading on the adsorption of Cd(II) ions was investigated by differentiating doses of sorbents (5, 7.5, and 10 g/dm^3^) while retaining all other parameters such as solution concentration 100 mg/dm^3^, solution pH 5, phase contact time 360 min and temperature 295 K constant. The effects of sorbent dosage on the removal of Cd(II) ions are presented in Fig. 1c, d. It can be noticed that the increase in dose of magnetic biochars reduces the sorption capacity from 15.42 to 8.93 mg/g for MBC1 and from 16.44 to 9.32 mg/g for MBC2. Therefore, the optimum value is equal to 5 g/dm^3^ of magnetic sorbents which was applied in the heavy metal ion sorption process.

### Effect of Initial pH

**Fig. 2:**
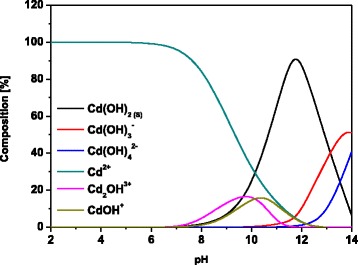
Speciation diagram for Cd(II)

Studies on the effect of pH are very important to optimize the sorption process. The value of pH affects the degree of ionization and the surface charge of the sorbent [31]. The influence of initial pH of the Cd(II), Co(II), Zn(II) and Pb(II) solutions on the sorption capacities of the sorbents was investigated by differentiating the initial pH from 2 to 6 and maintaining the other parameters and is shown in Fig. 1e, f. The presence of negatively charged groups on the surface of magnetic biochars allows sorption of positively charged Cd(II), Co(II), Zn(II) and Pb(II) ions [32]. Sorption of all metal ions at pH 2 is very low due to the presence of hydronium ions that occupy free places on the sorbent surface and excludes the possibility of metal ion binding. While the increase of pH will facilitate ion uptake [33], the equilibrium capacities of all metal ions increase and achieve the highest value at pH 5 (this pH value was selected as optimal for further research). Additionally, based on the speciation diagram (Fig. 2) for the pH values 5.0 and 6.0 Cd^2+^ was predominant.

### Adsorption Isotherms

To understand interactions between the metal ions and the sorbent is important to calculate the parameters of isotherms and correlation coefficients. The adsorption equilibrium data for Co(II) and Zn(II) ions were calculated using the three equations of the Langmuir, Freundlich and Temkin isotherm models and are listed in Table 3. In Table 4, the isotherm parameters and correlation coefficients as a function of temperature for the adsorption of Cd(II) are presented. Figure 2a, b shows the Cd(II) adsorption isotherms and fitted models. Comparing the parameters of isotherms, it can be stated that the value of *R* ^*2*^ (>0.95) from the Langmuir isotherm is the highest indicating a good fit to the experimental data. The Langmuir isotherm model assumes monolayer adsorption and neglects interactions between the molecules of adsorbate [34, 35]. In addition, the values of *R*_*L*_ from 0 to 1 indicate favourable adsorption nature [36].

**Table 3:** Adsorption isotherm parameters and correlation coefficients for the adsorption of Co(II) and Zn(II) on MBC1 and MBC2

Isotherm model	Parameters	MBC1		MBC2	
		Co(II)	Zn(II)	Co(II)	Zn(II)
Langmuir ${\mathit{\mathsf{q}}}_{\mathit{\mathsf{e}}}=\frac{{\mathit{\mathsf{q}}}_{\mathsf{0}}{\mathit{\mathsf{K}}}_{\mathit{\mathsf{L}}}{\mathit{\mathsf{C}}}_{\mathit{\mathsf{e}}}}{\mathsf{1}+{\mathit{\mathsf{K}}}_{\mathit{\mathsf{L}}}{\mathit{\mathsf{C}}}_{\mathit{\mathsf{e}}}}$	*q*_*e,*exp_	28.55	34.11	29.40	35.40
	*q*_0_	27.91	34.41	29.82	35.27
	*K*_*L*_	0.029	0.045	0.024	0.080
	*R*_*L*_	0.412	0.309	0.452	0.199
	*R* ^2^	0.960	0.973	0.975	0.992
Freundlich ${\mathit{\mathsf{q}}}_{\mathit{\mathsf{e}}}={\mathit{\mathsf{K}}}_{\mathit{\mathsf{F}}}{{\mathit{\mathsf{C}}}_{\mathit{\mathsf{e}}}}^{\mathsf{1}/\mathit{\mathsf{n}}}$	*K*_*F*_	8.47	12.31	8.85	15.45
	1*/n*	0.182	0.157	0.177	0.133
	*R* ^2^	0.940	0.922	0.874	0.918
Temkin ${\mathit{\mathsf{q}}}_{\mathit{\mathsf{e}}}=\frac{\mathit{\mathsf{R}}\mathit{\mathsf{T}}}{{\mathit{\mathsf{b}}}_{\mathit{\mathsf{T}}}}\mathsf{ln}\left({\mathit{\mathsf{a}}}_{\mathit{\mathsf{T}}}{\mathit{\mathsf{C}}}_{\mathit{\mathsf{e}}}\right)$	*A*	7.507	90.862	10.645	959.645
	*B*	3.084	2.876	3.050	2.528
	*b*_*T*_	803.36	861.41	812.31	980.09
	*R* ^2^	0.875	0.854	0.832	0.896

**Table 4:** Adsorption isotherm parameters and correlation coefficients as a function of temperature for the adsorption of Cd(II) on MBC1and MBC2

System	Parameters	MBC1			MBC2		
		Temperature [K]					
		295	315	335	295	315	335
Langmuir	*q*_*e,*exp_	37.64	32.50	26.85	41.33	39.44	32.52
	*q*_0_	38.00	35.04	28.08	41.25	41.68	32.71
	*K*_*L*_	0.182	0.045	0.043	0.191	0.072	0.068
	*R*_*L*_	0.099	0.310	0.317	0.095	0.216	0.227
	*R* ^2^	0.999	0.952	0.993	0.990	0.982	0.994
Freundlich	*K*_*F*_	13.06	9.29	4.49	13.06	10.76	6.54
	1/*n*	0.204	0.225	0.332	0.187	0.249	0.304
	*R* ^2^	0.976	0.618	0.794	0.966	0.627	0.771
Temkin	*A*	37.542	7.590	0.796	102.432	5.344	1.646
	*B*	4.103	4.255	4.974	3.990	5.660	5.347
	*b*_*T*_	603.84	582.35	498.15	620.99	437.77	463.38
	*R* ^2^	0.983	0.698	0.917	0.986	0.631	0.897

### Thermodynamic Tests

The thermodynamic parameters were obtained by the sorption at different temperatures in the range 295–335 K and are calculated (Eqs. 2–5) and listed in Table 5. In contrast to some literature reports [22] with the increasing temperature, the equilibrium capacity decreases from 37.64 mg/g at 295 K to 26.85 mg/g at 335 K for Cd(II) sorption on MBC1 (Table 4). Simultaneously, the value of the equilibrium constant *K*_*L*_ decreases with the increasing temperature from 0.182 to 0.043 dm^3^/mg for MBC1. These results also demonstrate that Cd(II) ion sorption on magnetic sorbents would be more efficient at lower temperatures [35].

**Table 5:** Thermodynamic parameters for the sorption of Cd(II) ions on MBC1 and MBC2

Sorbent	*K*_*d*_			*∆H* ^0^	*∆S* ^0^	*∆G* ^0^		
	Temperature [K]					Temperature [K]		
	295	315	335			295	315	335
MBC1	0.1170	0.1120	0.0870	−5.87	−37.5	−11.60	−12.28	−12.37
MBC2	0.1352	0.1321	0.1167	−2.36	−24.2	−11.95	−12.71	−13.18

The negative values of enthalpy change reveal that Cd(II) sorption on the magnetic sorbents is an exothermic process. In addition, the value of *∆H* ^0^ in the range up to 40 kJ/mol evidences physical adsorption [37]. The increase in the interactions at the solid-solution interface and reduction of the degree of disorder lead to a negative values of entropy change [38, 39]. The negative values of free energy change in the range −20 to 0 kJ/mol for all temperatures point out that the ion sorption is spontaneous and also testy to the physical character of sorption [38]. The decreasing value of *∆G* ^0^ with the increasing temperature can be associated with more favourable sorption at lower temperatures. In addition, for the exothermic processes, the value of *K*_*d*_ decreases with the increasing temperature from 0.1170 to 0.0870 for Cd(II) sorption on MBC1.

### Regeneration of Spent Sorbent

Reducing the cost and toxicity of the wastes after sorption is possible by conducting the regeneration process [40]. In the regeneration, there are used, cheap and easily accessible desorbing agents such as solutions of acids [32], salts, alkalis and complexing agents [18].

**Fig. 3:**
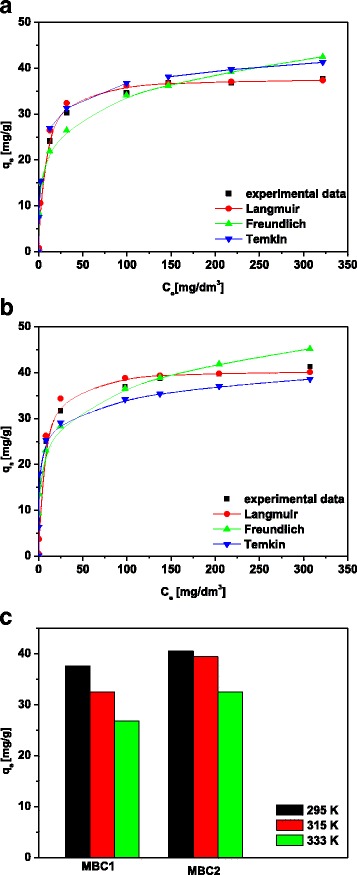
Isotherm data and fitted models for Cd(II) sorption on **a** MBC1 and **b** MBC2 and **c** effect of temperature on Cd(II) sorption on MBC1 and MBC2

In order to investigate the desorption action of Cd-loaded magnetic sorbents, distilled water and solutions of nitric acid(V) at the concentrations 0.1, 0.5, 1.0, 1.5, 2.0 and 5.0 mol/dm^3^ were applied. The use of distilled water resulted in the yield of 2.41%. The investigations carried out by Reguyal et al. [38] using deionized water proved that the desorption effectiveness is lower than 4% in the case of desorption of sulfamethoxazole-loaded magnetic biochar. Acidic desorbing agents have a higher capacity elution of the positively charged metal ions from the sorbent surface. This is due to the presence of hydronium ions which protonate the sorbent surface [41]. Of the concentrations used in the experiment, the best efficiency of desorption of Cd-loaded MBC2 equal to 97.09% is accounted for 0.1 mol/dm^3^ HNO_3_ (Fig. 3a). With an increase in nitric acid(V) concentration, the desorption percentage slightly decreases. For this reason, for further studies, 0.1 mol/dm^3^ HNO_3_ was used for desorption kinetics. From Fig. 3b, it can be stated that with an increase of the contact time, efficiency of desorption increases. After the time about 180 min, the percentage of desorption Cd-loaded MBC1 and MBC2 was constant.

### Characterization of the Sorbents

**Fig. 4:**
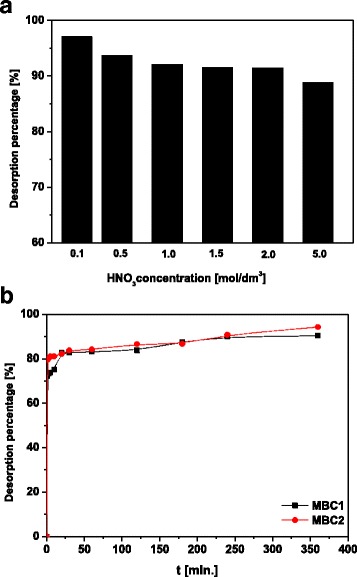
**a** Elution of Cd(II) from metal-loaded MBC2 using HNO_3_ at the concentrations in the range 0–2 mol/dm^3^ and **b** effect of the phase contact time on Cd(II) desorption on metal-loaded MBC1 and MBC2 using 0.1 mol/dm^3^ HNO_3_

Changes in the vibration of functional groups in the two types of magnetic biochar before and after Co(II) sorption are demonstrated in the FTIR spectra in Fig. 4a, b. The broad bands in the range of 3300 to 3500 cm^−1^ indicate the presence of hydroxyl groups either free or associated in groups –COOH and –CHO. The sharp peak at 3740 cm^−1^ in MBC1 before sorption can be assigned to OH group vibrations in mineral matter [42, 43]. The peaks in the range 2000 to 2380 cm^−1^ correspond to –C≡C– triple bond of alkynes. Also in this wave number range, vibrations of the groups of amines appear [43]. The bands of a wave number from 1395 to 1628 cm^−1^ testify to the presence of C=O and C=C aromatic vibrations in ring and C=O stretching of ketone and carboxyl groups [37, 44, 45] The presence of C–H aromatic branching results in the bands at about 980 cm^−1^ [46]. The peak at about 680 cm^−1^ in magnetic biochar is evidenced by the presence of Fe-biochar bonds. The disappearance of a sharp band at 3740 cm^−1^ after Co(II) sorption on MBC1 and moving the vibration derived from carboxyl groups causes that the OH and C=O groups are involved in formation of the bonds between the biochar surface and Co(II) ions [44, 47].

**Fig. 5:**
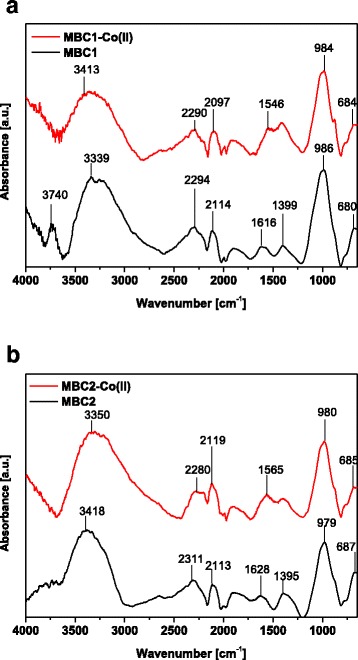
FTIR spectra of **a** MBC1 and **b** MBC2 before and after the sorption of Co(II)

Figure 5a, f presents the SEM images of MBC1 and MBC2 at different magnifications ×10000 (a, b), ×3500 (c, d) and ×100 (e, f). It can be concluded that the sorbent structure is irregular and the nanoparticles Fe(0) are well dispersed on the surface. Based on the images magnified ×100, it can be seen that the smaller are particles in MBC2, the better sorption properties are obtained.

**Fig. 6:**
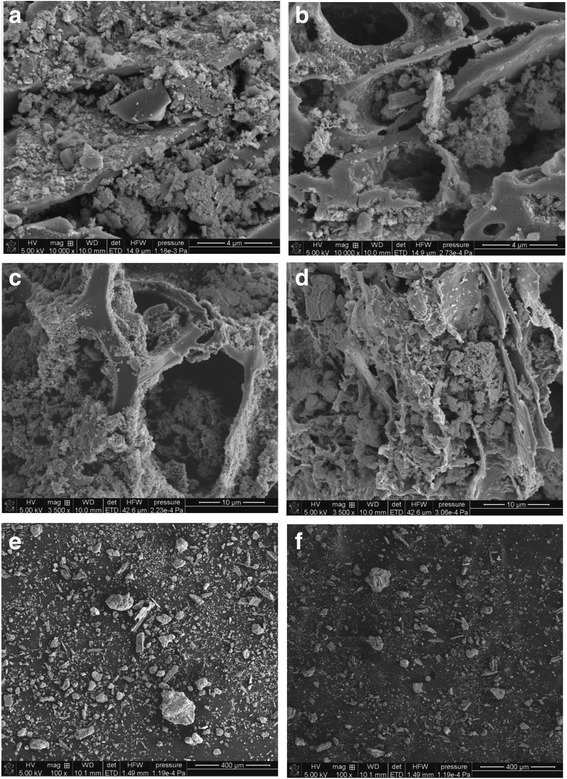
SEM images of MBC1 (**a**, **c**, **e**) and MBC2 (**b**, **d**, **f**) at different magnifications

The XRD analysis is applied to study the ordered structures present in biochars [48]. Figure 6 shows the X-ray diffraction analysis of MBC2 after Cd(II), Co(II), Zn(II) and Pb(II) ion sorption. The main peaks of the highest intensity at 2*Ɵ* = 26.80 and those at 2*Ɵ* = 20.58 confirm the silica (quartz) presence. The peaks indicating the presence of carbon appear at 2*Ɵ* = 29.48 which is due to the presence of calcium carbonate (calcite) and at 2*Ɵ* = 30.90 due to the calcium magnesium carbonate (dolomite) presence. The peaks at 2*Ɵ* = 44.80 indicate that Fe(0) occurs in the structure of magnetic biochar. These results are consistent with the previous literature reports [22, 48, 49].

**Fig. 7:**
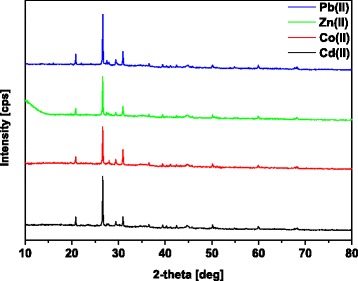
XRD analysis of MBC2 after Cd(II), Co(II), Zn(II) and Pb(II) ion sorption

The analysis of MBC2 spectrum after the Cd(II) ion sorption by means of X-ray photoelectron spectroscopy shows that the sorbent surface is composed of the atoms C, O, Fe, Mg, Si, Al, P, Ca, Cd and K (Fig. 7). This confirms the effectiveness of biochar modification by iron.

The XPS analysis also confirmed the presence of hydroxyl, carboxyl and carbonyl groups in the MBC2 samples (Table 6). The presence of C–C bonds in the aromatic ring can act as *π* donors in the process of ion sorption. In addition, the precipitation process of CdCO_3_ and Cd(OH)_2_ on the magnetic biochar surface also occurs. The presence of iron at various degrees of oxidation on the sorbent surface indicates an incomplete reduction to Fe^0^. Therefore, the modification process still requires further optimization [2].

**Table 6:** Fitted C 1s, O 1s, Cd 3d, and Fe 2p peak parameters deduced from the XPS analysis for MBC2 after the Cd(II) ion sorption

Region	Peak [eV]	Assignment	Atomic content [%]
C 1s	284.5	C=C sp^2^	72.7
	286.2	C–OH, C–O–C	7.8
	287.3	C=O	4.3
	288.5	COO–	6.1
	289.6	Carbonates	4.9
	291.2	*π* → π*	4.2
O 1s	530.2	Metal oxides	32.7
	531.4	C=O	35.8
	532.3	O=C–OH, C–OH	22.7
	533.3	C–O–C	8.7
Cd 3d	405.6	CdCO_3_, Cd(OH)_2_, –OCdOH	100
	412.4	–	–
Fe 2p	708.6	Fe(0)	2.4
	709.6	Fe(II)	4.9
	710.6		2.4
	714.9		2.4
	710.6	Fe(III)	31.9
	711.6		24.0
	712.6		16.0
	713.6		8.0
	719.5		8.0

**Fig. 8:**
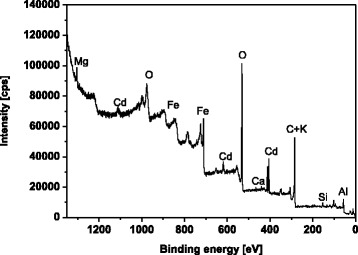
XPS full spectra of MBC2 after Cd(II) sorption

In Fig. 8a, b, the thermogravimetric and derivative thermogravimetric curves for MBC1 and MBC2 are shown. The TG curve presents the percentage weight loss of the sorbent and the DTG curve demonstrates the temperature at which the weight changes are most evident. The heating process is conducted up to 1273 K with the heating rate 283 K/min. From the curves, it can be concluded that the first stage of thermal degradation occurs in the range of 323–473 K which is associated with the loss of moisture. The subsequent degradation stages proceeded up to a temperature of 1073 K which is related with decomposition of hemicellulose, cellulose and lignin. The total weight loss (35%) took place up to a temperature of 1273 K [14, 50]. For both modifications, similar curves of thermal degradation were obtained.

**Fig. 9:**
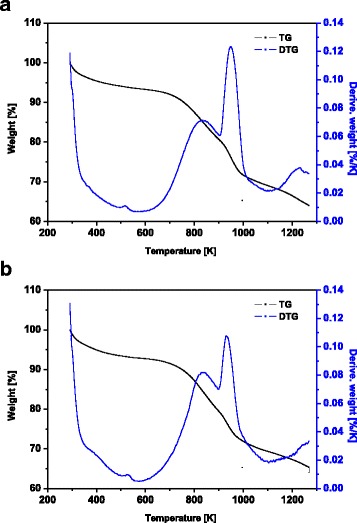
TG/DTG curves of **a** MBC1 and **b** MBC2

The point of zero charge pH_PZC_ is defined as the point at which the surface charge equals zero. The isoelectric point pH_IEP_ is defined as the point at which the electrokinetic potential equals zero. Figure 9a presents a course of potentiometric titration of dispersion of BC at the constant solid to liquid ratio and at three different concentrations of NaCl, with pH_PZC_ = 10.5. The zeta potential value for all studied concentrations in the whole pH range for the BC/electrolyte system is negative and independent of the electrolyte. pH_IEP_ is below 3.

**Fig. 10:**
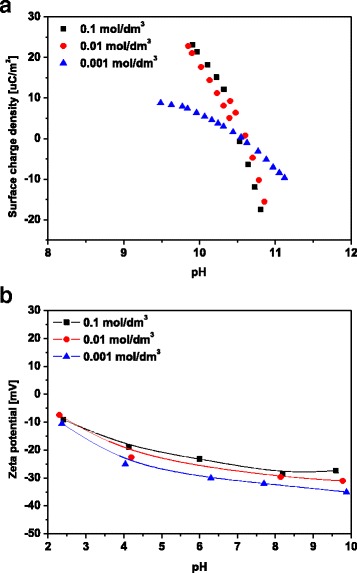
**a** Surface charge of biochar in aqueous solution of NaCl as a function of pH and **b** diagram of biochar potential zeta dependence on pH value in aqueous NaCl solutions

Knowledge of the zeta potential value enables prediction of colloidal system stability. The zeta potential allows to determine electrostatic interactions among the colloidal particles, and thus, it can be referred to the colloidal system stability. The BC zeta potential allows characterization of the double electrical layer at the BC/electrolyte solution interface. The particles BC in the electrolyte possess the electrical charge and the zeta potential allowing determining part of the charge in the double diffusion layer. The results are presented in Fig. 9b. The plot of the zeta potential dependence indicates that the value of the zeta potential changes insignificantly with the pH increase for a given concentration of the electrolyte. The dependence of the zeta potential in the pH function allows to assume that pH_IEP_ has the value <2 and is lower than the pH_PZC_ value, as the zeta potential depends also on the part of the surface charge which is affected by BC ions adsorbing or desorbing on the crystal lattice (Fig. 10). For the electrostatically stabilized systems, the higher the zeta potential is, the more probable the dispersion stability is. For the water systems from −30 to 30 mV, the border for stability of dispersion and its lifespan is assumed. With the rise of absolute value of the zeta potential, colloidal particles possess good dispersion properties, simultaneously with the rise of electrostatic repulsion which is visible for the examined BC/NaCl.

## Conclusions

Magnetic biochar nanocomposites were synthesized. Two types of modifications MBC1 and MBC2 for the removal of Cd(II), Co(II), Zn(II) and Pb(II) ions from aqueous solutions were used. Based on the research, it can be concluded that the operating parameters such as phase contact time, initial concentration of metal ions, dose of the sorbent solution pH and temperature play an important role in the sorption process. Additionally, on the basis of the PSO and Langmuir isotherm models, it can be seen that the higher affinity for the above-mentioned heavy metals is exhibited by MBC2. Therefore, a higher content of a reducing agent has a beneficial effect on the magnetic properties of sorbent. Desorption with 0.1 mol/dm^3^ HNO_3_ gives a yield of 97.09% and provides an easy regeneration of the obtained sorbents. The XRD analysis confirmed the presence of Fe(0) in the structure of the magnetic biochars. Following from the presented TG/DTG data, the total weight loss of sorbent up to a temperature 1273 K is about 35%. Both XRD and XPS analyses confirm the presence of iron on the biochar surface which proves successful modification. The point characteristics of the double layer for biochar are pH_PZC_ = 10.5 and pH_IEP_ <3.
