# Erratum to: Investigations of Heavy Metal Ion Sorption Using Nanocomposites of Iron-Modified Biochar

**DOI:** 10.1186/s11671-017-2229-z

**Published:** 2017-07-26

**Authors:** D. Kołodyńska, J. Bąk, M. Kozioł, L. V. Pylychuk

**Affiliations:** 1 Department of Inorganic Chemistry, Faculty of Chemistry, Maria Curie-Skłodowska University, M. Curie Skłodowska Sq. 2, 20-031 Lublin, Poland; 2 Department of Organic Technologies, New Chemical Syntheses Institute, Al.Tysiąclecia Państwa Polskiego 13A, 24-110 Puławy, Poland; 3 Nanomaterials Department, Chuiko Institute of Surface Chemistry of the National Academy of the Sciences of Ukraine, General Naumov Str, Kyiv, 03-164 Ukraine

## Erratum

In the original publication [1] the author name L. V. Pylychuk had a spelling error and one sentence under paragraph “Effect of Initial pH” consisted an error.

Incorrect name: L. V. Pylypchuk

Correct name: L. V. Pylychuk

Wrong sentence:

Additionally, based on the speciation diagram (Fig. 2) for the pH values 5.0 and 6.0 Cd2+, Cd(II) was predominant.

Should have read:

Additionally, based on the speciation diagram (Fig. 2) for the pH values 5.0 and 6.0 Cd2+ was predominant.

The original article has been updated to rectify these errors.
